# High-Throughput Amplicon-Based Copy Number Detection of 11 Genes in Formalin-Fixed Paraffin-Embedded Ovarian Tumour Samples by MLPA-Seq

**DOI:** 10.1371/journal.pone.0143006

**Published:** 2015-11-16

**Authors:** Olga Kondrashova, Clare J. Love, Sebastian Lunke, Arthur L. Hsu, Australian Ovarian Cancer Study (AOCS) Group, Paul M. Waring, Graham R. Taylor

**Affiliations:** 1 Department of Pathology, University of Melbourne, Melbourne, VIC, Australia; 2 Peter MacCallum Cancer Centre, Melbourne, VIC, Australia; 3 Centre for Cancer Research, University of Sydney at Westmead Millennium Institute, and Departments of Gynaecological Oncology, Westmead Hospital, Sydney, New South Wales, Australia; 4 QIMR Berghofer Medical Research Institute, Brisbane, QLD, Australia; CNR, ITALY

## Abstract

Whilst next generation sequencing can report point mutations in fixed tissue tumour samples reliably, the accurate determination of copy number is more challenging. The conventional Multiplex Ligation-dependent Probe Amplification (MLPA) assay is an effective tool for measurement of gene dosage, but is restricted to around 50 targets due to size resolution of the MLPA probes. By switching from a size-resolved format, to a sequence-resolved format we developed a scalable, high-throughput, quantitative assay. MLPA-seq is capable of detecting deletions, duplications, and amplifications in as little as 5ng of genomic DNA, including from formalin-fixed paraffin-embedded (FFPE) tumour samples. We show that this method can detect *BRCA1*, *BRCA2*, *ERBB2* and *CCNE1* copy number changes in DNA extracted from snap-frozen and FFPE tumour tissue, with 100% sensitivity and >99.5% specificity.

## Background

Detection of copy number variations (CNVs) in cancer receives less attention than detection of mutations, despite CNVs being relatively common, and having an important role in tumour initiation, progression, and treatment response in multiple cancer types. Focal somatic gene amplifications are also important targets for approved therapies, such as for trastuzumab, lapatinib, in *ERBB2* (*HER2)* amplified breast cancer or gastroesophageal cancer [1], and for potential therapies in genes such as *MET* and *CCNE1* in multiple cancers [2,3]. Similarly, loss of function due to germline or somatic deletions in tumour suppressor genes may confer drug sensitivity, such as that of high grade serous ovarian cancer with *BRCA1* or *BRCA2* mutations, to the PARP inhibitor olaparib [4].

The current methods for CNV detection in a diagnostic setting include qPCR, DNA microarrays, *in situ* hybridization (ISH) and MLPA [5–8]. Each relies on a different technology and have their own advantages and disadvantages. However, to date, there are no methods to accurately and reproducibly measure low copy number amplifications for diagnostic purposes using next-generation sequencing (NGS)–a technology that is being increasingly used in diagnostics for mutation detection, from small gene panels, to exome sequencing. Development of a method, which utilises NGS for CNV detection, would allow testing for mutations and CNVs to be performed on the same platform and at the same time, thus, simplify the laboratory work, reduce the cost of testing, and allow for high-throughput screening.

Here we present an amplicon-based method for CNV detection called MLPA-seq, based on the original MLPA assay, applied to a NGS technology. This method, unlike traditional MLPA, does not require amplified products to be of different size for capillary electrophoresis separation, consequently, the number of probe pairs mixed in a single reaction is no longer limited to 50. In this study, we applied the MLPA-seq for screening of ovarian cancer, focusing on the genes commonly amplified or deleted.

## Materials and Methods

### Sample selection

DNA extracted from blood samples from 12 de-identified individuals with germline *BRCA1* or *BRCA2* deletions and duplications (exonic or whole gene) were obtained from PathWest, Western Australia, and the Australian Ovarian Cancer Study (AOCS) [9].

Breast cancer samples with >50% tumour purity and known *ERBB2* amplifications (6 FFPE tumour samples), and high grade serous ovarian cancer samples with >50% tumour purity and *CCNE1* amplifications (5 FFPE tumour and 3 snap-frozen tumour samples from 6 patients), were obtained from the Royal Melbourne Hospital and the Victoria Cancer Biobank (VCB), respectively. *ERBB2* amplifications were detected using INFORM HER2 Dual ISH DNA Probe Cocktail Assay (Ventana, Tucson AZ), and *CCNE1* amplifications were detected using chr19q12 ISH assay (Ventana).

This project was approved by and conducted under Melbourne University Human Research Ethics Committee project #1238381. Written informed consent for use of samples in future research was previously obtained from individuals enrolled through AOCS and VCB. Consent was waived by the ethics committee for patient samples received from diagnostic laboratories (PathWest, Western Australia and the Royal Melbourne Hospital).

### Sample preparation

DNA from macrodissected FFPE tumour tissue, snap-frozen tumour, and blood samples was extracted using QIAamp FFPE Tissue kit (Qiagen, Catalog No. 56404) and QIAamp DNA Mini Kit (Qiagen, Catalog No. 51304), respectively, according to the manufacturer’s protocol.

All DNA samples were ethanol precipitated and resuspended in low TE (10mM Tris, 0.1mM EDTA) buffer at pH8. DNA samples were quantified using Qubit dsDNA HS Assay Kit (Life Technologies), and DNA samples from FFPE tumour tissue were also assessed for DNA fragmentation using an in-house developed multiplex-PCR assay adapted from the GAPDH assay described by van Beers *et al*., targeting *GAPDH* gene to amplify fragments of 100 bp, 200 bp, 300 bp and 400 bp. [10]. The amplified products were then assessed on a 2% agarose gel, where the presence of different products indicated various DNA quality: samples with faint or no 200 bp product were assigned a ‘very poor’ status, samples with clearly visible 200 bp product were assigned a ‘poor’ status, samples with clearly visible 300 bp product were a assigned a ‘moderate’ status, while samples with all four visible products were assigned a ‘good’ status.

### Probe selection

The assay was designed to target 11 genes commonly amplified or deleted in ovarian cancer (Table 1). *ITIH5* (10p14) and *ANXA7* (10q22) and *HTRA1* (10q26) were targeted to assess if the deletion of *PTEN* (10q23) is localised, since they are located upstream and downstream from *PTEN*, while *PTENP1* (9p11), a highly homologous pseudogene of *PTEN*, was targeted to confirm copy number status of *PTEN*. In addition, 7 other genes (*CFTR*, *GCH1*, *JAG1*, *OPTN*, *GPC3*, *PANK2* and *FLCN*) were targeted as reference controls for copy number normalisation.

**Table 1:** Targeted gene list, transcripts and design features.

Target	Transcript	Frequency in High-Grade Serous Ovarian Carcinomas	Frequency in Other Ovarian Cancer Subtypes	Target Region	CNV Event	Number of Probes
*BRCA1*	NM_007294.3	0.59% [9]	-	All exons	Deletion (whole gene or exonic)	52 (26 used for confirmation)
*BRCA2*	NM_000059.3	0.35% [9]	-	All exons	Deletion (whole gene or exonic)	64 (33 used for confirmation)
*PTEN*	NM_000314.4	7% [11]	20–30% [12,13]	All exons	Deletion (whole gene or exonic)	20
*ERBB2*	NM_001005862.2	3.1% [11]	15–20% [14]	Exons 7 & 13	Amplification	2
*MYC*	NM_002467.4	34.2% [11]	-	Exon 3	Amplification	1
*MET*	NM_001127500.1	-	6% [15]	Exon 4	Amplification	1
*CCNE1*	NM_001238.2	22.7% [11]	-	Exons 6 & 11	Amplification	2
*NF1*	NM_000267.3	8% [11]	-	Exon 26	Deletion	1
*RB1*	NM_000321.2	7.8% [11]	-	Exon 6	Deletion	1
*AURKA*	NM_003600.2	27.6% [15]	27.6% [16]	Exon 10	Amplification	1
*EMSY*	NM_020193.4	7.9% [11]	-	Exon 16	Amplification	1
*PTEN* deletion confirmation				4 genes		4
Reference	-	-	-	7 genes	-	7
					**Total Number of Probes**	**157**

The probe locations and sequences were obtained from publically available MLPA probe designs (MRC-Holland, www.mlpa.com). Nextera adapter overhangs were added to each probe to allow addition of Nextera XT indexes (Illumina) during the PCR reaction. Probes were ordered from IDT (Ultramers with a phosphate group on 5’ end of each the right probe oligo). Two probe mixes were used, one for detection (98 probes), and one for confirmation (100 probes), listed in S1 Table.

### MLPA-seq library preparation

**Fig 1:**
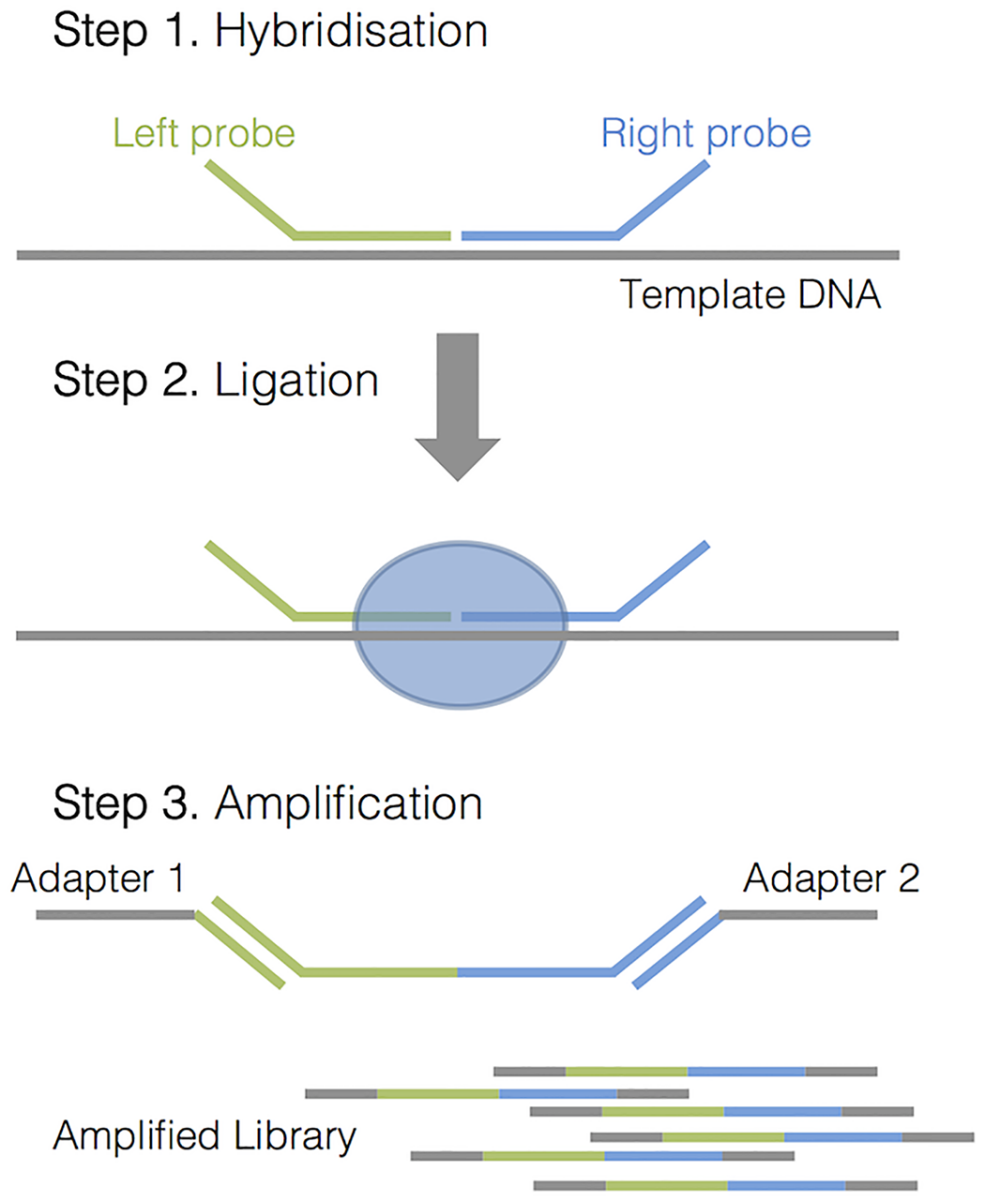
An outline of steps required for library preparation in the MLPA-seq.

Library preparation included three major steps, which are outlined in Fig 1. In each experiment, two separate libraries (detection probe mix and confirmation probe mix) were prepared for each sample, including normal control (without CNVs) and NTC.

#### Hybridization reaction

5 μL of 4 ng/ μL DNA sample was denatured for 5 minutes at 98°C and then cooled to room temperature. 1.5 μL of MLPA buffer (MRC-Holland) and 1.5 μL of probe mix (each probe at 0.3 nM concentration) was added to each denatured sample, and incubated for 1 minute at 95°C, then for 15 hours at 60°C.

#### Ligation reaction

3 μL Ligase-65 buffer A (MRC-Holland), 3 μL Ligase-65 buffer B (MRC-Holland), 25 μL ddH_2_O, and 1 μL of Ligase-65 enzyme (MRC-Holland) were added directly to the hybridization reaction, and incubated for 15 minutes at 54°C, followed by 5 minutes at 98°C for heat inactivation of the Ligase-65 enzyme, and then paused at 15°C.

#### Indexing PCR reaction

10 μL of ligation reaction was added to 5 μL of 5× Q5 Reaction buffer (NEB), 3.75 μL of dH2O, 1 μL of each Nextera XT index (unique i5 and i7, Illumina), 2 μL of dNTPs (2.5 mM each, Bioline), and 0.25 μL of Q5 Hot Start High-Fidelity DNA Polymerase (NEB). The PCR was performed as follows: 30 seconds at 98°C for initial denaturation, followed by 25 cycles of 10 seconds at 98°C, 15 seconds at 63°C, and 20 seconds at 72°C, followed 2 minutes at 72°C for final extension.

The libraries were cleaned using standard Agencourt AmpureXP beads (Beckman Coulter) procedure with DNA to bead ratio of 1:0.9, and eluted in 20 μL of ddH_2_O.

Library quality control was performed by analysing the library fragment size distribution on a 2% agarose gel, and molar concentrations were calculated from the concentrations obtained by using the Qubit dsDNA HS Assay Kit. Libraries were normalised to 2 nM concentration, then pooled and denatured according to the manufacturer’s instructions (Preparing Libraries for Sequencing on the MiSeq, #15039740, Revision D, Illumina). They were then sequenced using MiSeq v2 300-cycle kit (Illumina) at 15 pM final concentration according to the MiSeq System User Guide (#15027617, Revision M, Illumina), with 150 separate reaction libraries per run.

### Analysis

Analysis of sequencing data was performed using AmpliVar Genotyping workflow [17], with a suspects file containing 20 bases of the middle sequence of each probe pair, and the Nextera adapter option specified. A custom R script (MLPAseq-Reporter) was developed for analysis of the data output from the AmpliVar workflow, which produced a report text file, as well as the graph plotting mean ratios for each exon (open source script, available on GitHub: https://github.com/okon/MLPAseq-Reporter).

Probes were first normalised within each library (Eq 1), where each raw coverage value was divided by the mean of all raw coverage values (Eq 2), excluding probes covering genes with potential amplifications (*CCNE1*, *EMSY*, *ERBB2*, *MET*, *MYC*, and *AURKA*) in that library, providing a depth-normalised value for each probe (*x’*_*k*_). CNV ratios (*x”*_*k*_) were then calculated for each probe (Eq 3), by dividing each depth-normalised probe value by the mean of all depth-normalised values for that probe in the accumulated control samples. Mean and standard deviation of the CNV ratios for each targeted exon (*x”*_*ij*_) were calculated. Probes for each targeted exon included detection and confirmation probes from two separate libraries, as well as different probes from one library that covered one exon (e.g. *BRCA1*, exon 12, probes b and c from confirmation mix).

$$x\prime_{ijk}=\frac{x_{ijk}}{x_{i}}$$ (1)

$$X_{i}=\frac{1}{\sum N_{j}}\sum_{j=1}^{j}\sum_{k=1}^{N_{j}}x_{ijk}\;(j\;\notin \text{ amplification exons})$$ (2)

$$x\prime \prime_{ijk}=\frac{x\prime_{ijk}}{\bar{x}\prime_{ijk}}\;(i\;\in \text{ control samples from multiple runs})$$ (3)

*i* = each sample

*j* = each exon

*k* = each probe

*N*_*j*_ = number of probes in exon *j*

*j* = number of exons covered by assay, excluding amplification exons

A summary report was produced for each sample, that stated a mean probe coverage for each library, the total number of aligned reads on target, a mean coverage for the no template control, and a mean ratio and standard deviation for each exon analysed in the sample (S2 Table).

## Results

### Definition of copy number variation

The ratios ranging from 0.7 to 1.3 were observed as part of normal variation, therefore targeted regions with such ratios were not considered to be amplified or deleted. If the observed mean ratios for the targeted regions were greater than 1.3 in blood and the corresponding tumour sample, then they were reported as a germline duplication, while if the increased mean ratios were only observed in the tumour sample, but not in blood, they were regarded as having a somatic amplification. To avoid calling false positives due to possible genomic instability in tumour samples, the amplifications were only called when the mean ratios were above 1.5 (average gene copy number above 3). The targeted regions with mean ratios between 0.3 and 0.7 were regarded as having a heterozygous deletion (average gene copy number of around 1). If detected in blood and the corresponding tumour sample, they were assigned a germline status, and if detected only in tumour, they were assigned a somatic status. Tumour samples with targeted regions with mean ratios of 0.3 or less were reported as a homozygous deletion (0 gene copies) resulting from a germline heterozygous deletion with loss of heterozygosity (LOH) of the other allele.

### Overall performance

**Fig 2:**
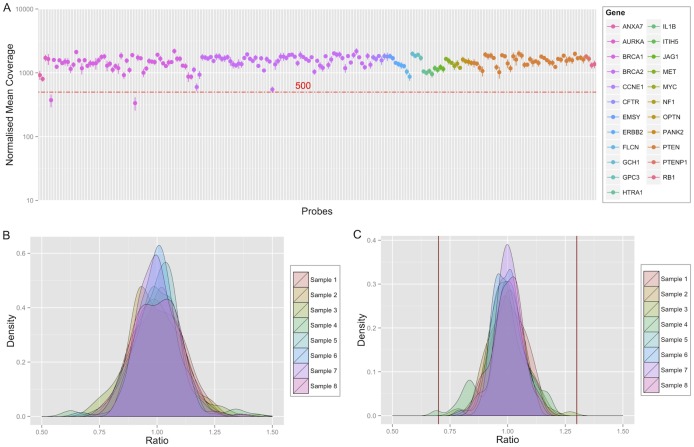
Coverage and calculated copy number ratios in the control samples (n = 8). (A) Mean probe coverage in the control samples, normalised to compensate for library indexing differences. The error bars represent standard deviation. The horizontal line at 500x coverage represents the minimum raw probe coverage for optimal results. (B) Distribution of calculated probe ratios in the control samples. The ratio represents observed over expected copy number for each probe, with 1 equal to normal copy number. (C) Distribution of combined ratios for each targeted exon in the control samples. The vertical lines represent 0.7 to 1.3 range of ratios considered normal.

Libraries with up to 100 probe pairs mixed in a single reaction were prepared, and up to 198 probe pairs for one sample were sequenced in the same sequencing run. Up to 75 samples (150 separate reactions) were combined in a single sequencing run to meet the required 1000-fold median probe coverage. The normalised target coverage was reproducible and consistent without any prior optimisation of input probe concentrations, with only two probe pairs falling below 500-fold coverage (Fig 2A). Calculated ratios for each probe in the control samples had a normal distribution with standard deviation of 0.103 (Fig 2B), which decreased to 0.072 (Fig 2C) after combining the probe values for each exon. One control sample, with median coverage below required (856-fold), had a ratio outside of the pre-defined normal range of 0.7–1.3.

### Limit of Detection

To estimate the limit of detection, DNA from a blood sample with a heterozygous germline *BRCA1* deletion (exons 1–23) was mixed with DNA from a blood sample with a heterozygous germline duplication of exon 12 in *BRCA1*, at 1:1, 1:2 and 2:1 ratios. Mixing of these samples at different proportions was expected to result in copy number ratios of 0.75, 0.67 and 0.83 for *BRCA1* exons 1 to 23, with the exception of exon 12, which was expected to result in copy number ratios of 1.17, 1.00 and 0.83.

**Fig 3:**
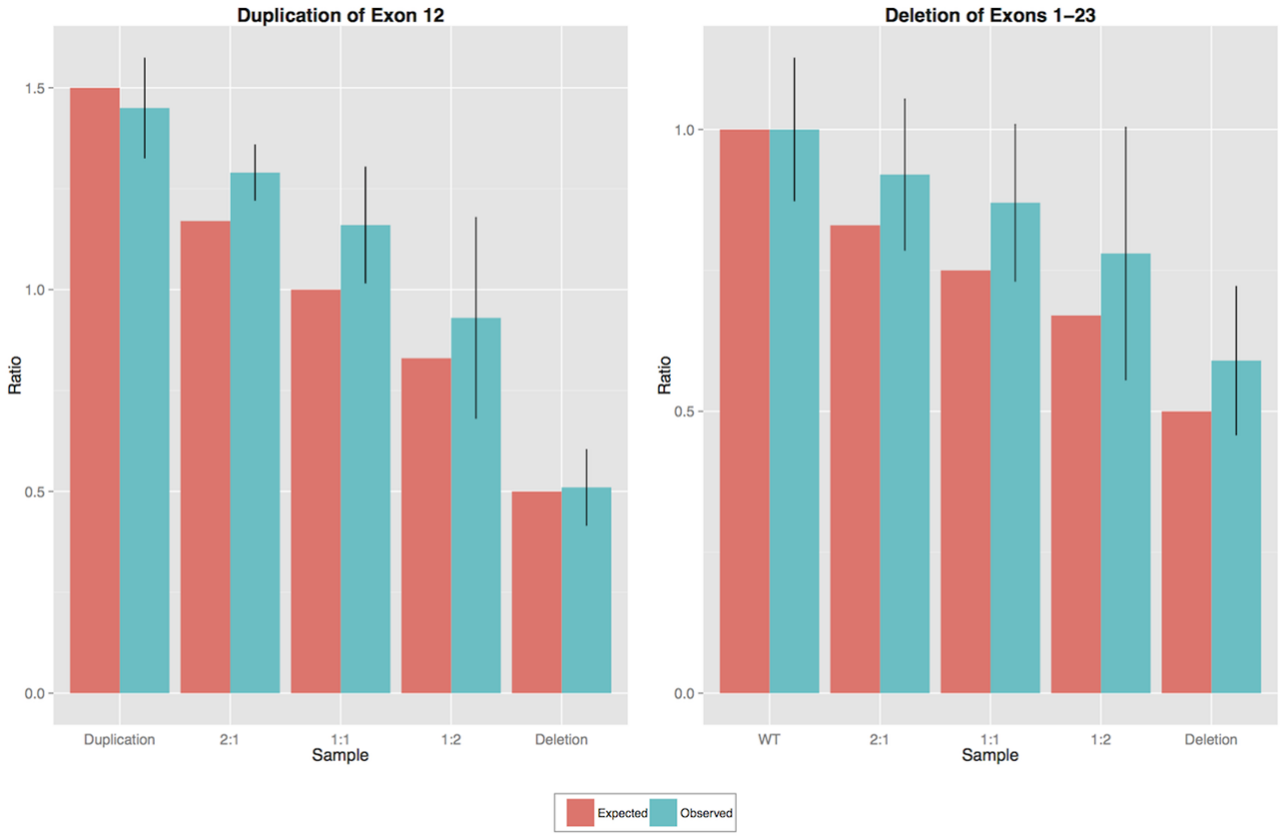
Observed vs. expected copy number ratios for two mixed samples, one with *BRCA1* exon 12 duplication and another with *BRCA1* exons 1–23 deletion. The error bars represent standard deviation of probe ratios.

The heterozygous *BRCA1* deletion of exons 1–23 and duplication of exon 12 were detected in unmixed samples, however, while there was a trend of decreasing ratios observed in all mixed samples for *BRCA1* exons 1–23 (excluding 12), the definitive copy number changes could not be determined with confidence in any of the mixed samples (Fig 3).

For samples containing a germline heterozygous deletion or duplication, the limit of copy number detection was determined to be one copy change regardless of the tumour purity. Tumour purity, which is the proportion of neoplastic versus all cells within the tumour, affected the ability to detect somatic copy number alterations. In tumour samples, the expected limit of detection of somatic deletions was one copy (heterozygous) for samples with 100% purity and two copies (homozygous) for samples with 50% tumour cell content.

### Sensitivity and Specificity

The analytical sensitivity of the MLPA-seq for detection of focal amplifications was tested in 14 snap-frozen tumour or FFPE tumour samples from 12 tumours with known *CCNE1* or *ERBB2* amplifications, originally detected with ISH assays. All known focal amplifications were detected. There was a moderate concordance of gene copy number as determined by ISH (Table 2). The ability to detect focal amplifications was unaffected by DNA sample quality as all focal amplifications were detected in both samples with good and poor quality DNA.

**Table 2:** Analytical sensitivity for detection of somatic amplifications of *CCNE1* and *ERBB2* genes in tumour samples—concordance between ISH assay and results from the MLPA-seq.

Sample Number	Sample Type	DNA quality^	Estimated Tumour Purity (%)	Gene	ISH: Mean number of copies	ISH: Level of amplification~	Developed Method: Mean number of copies ± SD	Developed Method: Level of amplification~	Level of amplification concordance
1	Frozen	Very good	NA	*CCNE1*	10.8*	medium	8.35 ±0.13	medium	concordant
2	FFPE	Moderate	70	*CCNE1*	10.8	medium	7.95 ±2.93	medium	concordant
3	FFPE	Moderate	80	*CCNE1*	4	low	3.16 ±0.71	low	concordant
4	FFPE	Good	95	*CCNE1*	3.48	low	6.6 ±0.62	medium	discordant
5	Frozen	Very good	NA	*CCNE1*	3.48*	low	5.63 ±0.18	low	concordant
6	Frozen	Very good	NA	*CCNE1*	7.2*	medium	7.77 ±0.18	medium	concordant
7	FFPE	Poor	90	*CCNE1*	9.3	medium	3.58 ±0.54	medium	concordant
8	FFPE	Moderate	90	*CCNE1*	7.2	medium	6.28 ±1.56	medium	concordant
9	FFPE	Good	80	*ERBB2*	10.7	medium	14.82 ±0.48	high	discordant
10	FFPE	Good	60	*ERBB2*	12	medium	39.22 ±1.53	high	discordant
11	FFPE	Very poor	80	*ERBB2*	20.5	high	9.39 ±0.24	medium	discordant
12	FFPE	Very poor	50	*ERBB2*	21.7	high	25.27 ±0.07	high	concordant
13	FFPE	Good	90	*ERBB2*	23.4	high	13.39 ±0.55	high	concordant
14	FFPE	Good	90	*ERBB2*	24.4	high	26.3 ±1.1	high	concordant

*Only matched FFPE tissue tested.

^^^ Very poor—< 200 bp amplifiable fragments, Poor—~ 200 bp amplifiable fragments, Moderate—200–300 bp amplifiable fragments, Good—300–400 bp amplifiable fragments, Very good—> 400 bp amplifiable fragments.

^~^Level of amplification defined as: >3 and ≤6 copies—Low, >6 and ≤12 copies—Medium, >12 copies–High.

Twenty DNA samples extracted from 16 blood samples and 4 tumour samples from 17 patients with known germline *BRCA1* or *BRCA2* copy number events, originally detected by MLPA, were used to assess the analytical sensitivity for detection of germline exonic deletions and duplications by the developed MLPA-seq method. The deletions ranged in length from a single exon to 23 exons. All previously known deletions, and the single duplication were identified by the MLPA-seq. The variation in the mean number of copies in the tumour samples was correlated to the tumour purity (Table 3). Additional copy number events were detected in some of the screened tumour samples, which included *MYC* and *EMSY* amplifications and *PTEN* exonic deletions (S3 Table).

**Table 3:** Analytical sensitivity for detection of germline copy number changes in *BRCA1* and *BRCA2* genes in normal, tumour and ascites samples—concordance between MLPA and results from the MLPA-seq.

Sample Number	Sample Type	DNA quality^	Gene	MLPA: Interpretation	Developed Method: Interpretation	Developed Method: Mean number of copies ± SD
1	Blood	Very Good	*BRCA1*	het del exon 4	het del exon 4	1.04 ±0.11
2	Blood	Very Good	*BRCA1*	het del exons 1–22	het del exons 1–22	1.04 ±0.16
3	Blood	Very Good	*BRCA1*	het del exons 1–2	het del exons 1–2	1.12 ±0.38
4	Blood	Very Good	*BRCA1*	het del exons 20–21	het del exons 20–21	0.94 ±0.03
5	Blood	Very Good	*BRCA1*	het del exons 1–11	het del exons 1–11	1.05 ±0.13
6	Blood	Very Good	*BRCA1*	het del exon 7	het del exon 7	1.02 ±0.25
7	Blood	Very Good	*BRCA1*	het del exons 1–16	het del exons 1–16	1.03 ±0.17
8	Blood	Very Good	*BRCA1*	het del exon 23	het del exon 23	0.92 ±0.2
9	Blood	Very Good	*BRCA2*	het del exons 1–2	het del exons 1–2	1.12 ±0.14
10	Blood	Very Good	*BRCA1*	het del exon 19	het del exon 19	0.96 ±0.1
11	Blood	Very Good	*BRCA2*	het del exons 1–2	het del exons 1–2	1.15 ±0.08
12	Blood	Very Good	*BRCA1*	het del exon 7	het del exon 7	1 ±0.06
13	Blood	Very Good	*BRCA1*	het dup exon 12	het dup exon 12	2.89 ±0.25
14	Blood	Very Good	*BRCA1*	het del exons 1–23	het del exons 1–23	1.17 ±0.27
15	Frozen	Very Good	*BRCA1*	del exons 1–23*	hom del exons 1–23	0.43 ±0.21
16	FFPE	Very Poor	*BRCA2*	del exons 14–16*	hom del exons 14–16	0.2 ±0.08
17	Blood	Very Good	*BRCA1*	het del exon 19	het del exon 19	1 ±0.16
18	FFPE	Moderate	*BRCA1*	dup exon 12*	het dup exon 12	2.83 ±0.74
19	Blood	Very Good	*BRCA2*	het del exons 14–16	het del exons 14–16	1.08 ±0.2
20	Ascites	Very Good	*BRCA1*	del exon 3*	hom del exon 3	0.07 ±0.04

*Only matched blood sample tested.

^^^ Very poor—< 200 bp amplifiable fragments, Poor—~ 200 bp amplifiable fragments, Moderate—200–300 bp amplifiable fragments, Good—300–400 bp amplifiable fragments, Very good—> 400 bp amplifiable fragments.

Analytical specificity was evaluated by comparing the predicted copy number of exons without germline deletions or duplications in *BRCA1* or *BRCA2* genes in the above-mentioned 16 blood DNA samples. Out of 766 true negative exon regions, four exons had ratios above 1.3 (two samples with two non-contiguous exons each). These false positives were exon duplications in *BRCA2* (exon 12 and exon 2; exon 7 and 13), which have not been previously reported in literature and mutation databases[18,19]. The false positives were a consequence of the large heterozygous deletions of *BRCA1* (exons 1–16 and exons 1–23) in those two samples, since these exons were used as part of probe normalisation. The overall profile of ratios for the other probes was also increased. The calculated specificity for detection of exonic deletions and duplications was therefore >99.5%.

### Reproducibility and Robustness

**Fig 4:**
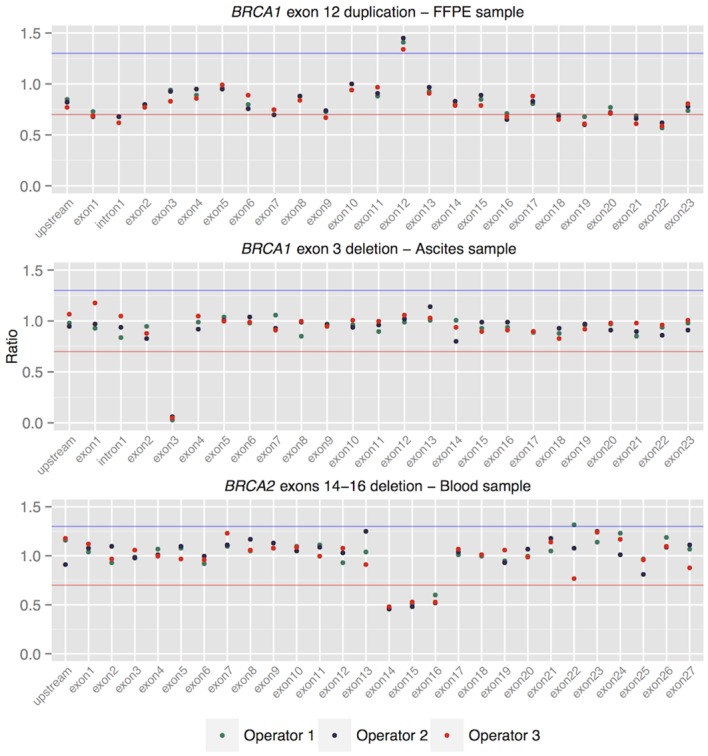
Ratios for *BRCA1*, *BRCA2* genes for three reproducibility samples, prepared by three operators. The red line indicates the lower limit of normal ratio variation (0.7), the blue line the upper limit of normal ratio variation coverage (1.3).

To evaluate reproducibility and repeatability, triplicate libraries were prepared for seven different samples with known germline or somatic CNV events, in single and separate experiments by different operators. Samples of different DNA quality and multiple sample types were selected, which represented the range of samples typically tested for CNVs. In the repeatability experiments, where replicates were prepared in single and multiple experiments by the same operator, the standard deviation of calculated ratios ranged from 0.000 to 0.117, irrespective of the sample type or DNA quality (S4 Table). In the reproducibility experiments, three different samples (blood, FFPE tumour and snap-frozen tumour) with known germline exonic *BRCA1* or *BRCA2* gene deletions or duplications were prepared in triplicates by three different operators. The observed ratios were reproducible for all replicates, even for the FFPE sample with poor DNA quality (Fig 4). Each deletion and duplication was successfully detected in every replicate.

Furthermore, to establish the minimum DNA input required for reproducible coverage, an experiment was performed, where 1 ng, 5 ng, 10 ng and 20 ng inputs of DNA derived from an FFPE sample with *ERBB2* and *MYC* amplifications with very poor DNA quality. Libraries with 1 ng input could not be successfully analysed due to multiple coverage dropouts, however, libraries with 5 ng and 10 ng inputs had similar coverage reproducibility to libraries with 20 ng input (S1 Fig). *ERBB2* and *MYC* amplifications were detectable at the same level in libraries with DNA inputs down to 5 ng.

### Analysis performance

To estimate the average speed of analysis, 84 FASTQ file pairs representing 84 separate reactions, with 187,559 read pairs on average, were analysed on a 64-bit Centos 6 operating system, Intel Xeon 2.20GHz processor using eight threads for AmpliVar Genotyping workflow, and a single thread for a custom developed R script. The whole fully automated analysis from raw FASTQ file input to graphical and text exon ratio output took 8 minutes 59 seconds, making it a very fast and easy approach to analyse the sequencing data directly from the MiSeq instrument.

## Discussion

While detection of single nucleotide variants and short indels using both amplicon and capture targeted NGS methods has been refined to be fit for diagnostic use, the detection of copy number changes and large deletions (exonic and whole gene) by NGS has yet to be optimised to the same level of sensitivity and specificity. Here we present a modified, inexpensive, accurate, precise, and reliable approach for detection of low-level copy number changes in blood or tumour samples, suitable for medical use without the need for confirmation by an orthogonal method.

The MLPA-seq is based on the traditional MLPA assay, where multiple probe pairs, with MiSeq compatible sequencing adapters on the ends, are hybridised to the template DNA, ligated and amplified using dual indexed adapters. Once the amplified probe pairs are sequenced and counted, the relative DNA copy number is estimated from the relative number of probe pairs detected. Since each reaction is uniquely indexed, multiple samples can be processed in a single sequencing run. In this study, we used Nextera XT adapters, which provide 384 different indexing combinations. This method was shown to be very sensitive and highly specific in detection of not only low level amplifications, but also single exon heterozygous deletions and duplications. Also, since this method utilises the traditional MLPA approach of amplifying short probe pairs (around 60 bp) instead of template DNA, it works well in samples with high DNA fragmentation, often seen in FFPE samples. The ability of processing FFPE samples is especially important in cancer diagnostic testing, as it is the most commonly used method for preservation of tumour samples.

The use of PCR amplicon dosage has been previously reported to detect deletions and duplications in *BRCA1* and *BRCA2* by Feliubadaló *et al* [20]. This method also reported too many false positive findings for routine diagnostic use, whereas at the method described here, based on the well-proven MLPA assay had specificity above 99.5%.

MLPA-seq overcomes the limitations of ISH assays, commonly used for CNV detection in diagnostic setting. Firstly, ISH assays have limited resolution of greater than 20 kb, thus are not suitable for exon-length CNV detection, while MLPA-seq can detect CNVs of probe-pair length. Furthermore, the analysis of ISH assays is labour-extensive, and cannot be scaled to high-throughput, high-multiplex testing.

Moreover, one of the advantages of MLPA-seq over the traditional MLPA assay is that probe pair lengths do not have to be variable for capillary electrophoresis separation, as the amplicons are identified by their sequence rather than length. This allows the method to include more than 50 probe pairs in a single reaction, which is the maximum number of probe pairs in traditional MLPA. This not only increases the number of possible targets but also eliminates amplification bias associated with larger fragments. Whilst it is possible to eliminate the size-selection component of MLPA using an array-based readout [21], the NGS approach is simpler and more adaptable. In this study, the method was tested with 100 probe pairs in a single reaction mix; however, more probe pairs can potentially be added if required. Since the raw coverage of amplified probe pairs is very uniform, no experimental optimisation is required when new probe pairs are added to the mix, making the method customisable and easily scalable. Furthermore, the read-count output from AmpliVar can be directly piped to a statistical package for dosage estimation, making the process highly automatable. In this study we used a custom R script (MLPAseq-Reporter) to generate our results.

Whilst the limit of detection for germline CNVs was established to be one copy, detection of somatic CNVs largely depends on tumour purity and genome stability. Since the MLPA-seq only detects relative copy number differences, tumour samples with large number of somatic CNVs may be difficult to analyse and interpret. One potential way to simplify analysis would be to add multiple control probes on each chromosome to get a broad overview of tumour ploidy and genome instability, which would aid in interpreting individual gene amplifications. One of the limitations of both the traditional MLPA assay and MLPA-seq is that probe hybridisation and ligation is sensitive to single nucleotide variations, insertions and deletions, especially, close to ligation site [8]. This can result in reduced region coverage, and thus be mistaken for a deletion. This possibility should always be considered in the analysis of single exon deletions, and confirmation by an independent method is often recommended [8]. To reduce the possibility of false positive deletions called in single exons of *BRCA1* and *BRCA2*, multiple probe pairs for each exon (located in different regions) were used for deletion confirmation.

In this study, the MLPA-seq was applied to ovarian cancer covering most genes, which are commonly amplified or deleted in this cancer. Coupled with a SNV and short indel detection method, this approach can be used as broad screening and stratifying tool for complex genetic diseases, such as ovarian cancer. This method could also be used to modify the methylation-specific MLPA (MS-MLPA) assay for simple targeted methylation analysis using next-generation sequencing.

## Conclusions

We have developed a NGS-based method for copy number detection with high accuracy and precision and rapid automated analysis ideal for medical use in the diagnostic setting. It works well in FFPE samples with highly fragmented DNA, as the amplified fragments are very short. It is also highly customisable and flexible in the number of targets that can be identified. The MLPA-seq method has multiple potential applications, including cancer diagnosis, and classification, inherited cancer risk assessment, prognostic estimation and patient selection and stratification for clinical trial enrolment and treatment.

## Supporting Information

S1 Fig Assessment of different DNA inputs (5ng, 10ng and 20ng) used for library preparation. An FFPE sample with very poor quality DNA was used for the assessment, with triplicate testing for lower DNA inputs (5ng and 10 ng).
(PDF)

S1 Table Design probe sequences and target genomic coordinates. *PTEN pseudogene, used for confirmation of true *PTEN* deletions. # Genes upstream and downstream from *PTEN*, used for assessing if *PTEN* deletions are localised. ^Reference genes, used for normalisation of copy number.
(XLSX)

S2 Table An example of a typical summary report produced by MLPA-seq Reporter software. (XLSX)

S3 Table Additional findings in tumour samples used for sensitivity analysis. (XLSX)

S4 Table Intra- and Inter-batch repeatability. ^ Very poor—< 200 bp amplifiable fragments, Poor—~ 200 bp amplifiable fragments, Moderate—200–300 bp amplifiable fragments, Good—300–400 bp amplifiable fragments, Very good—> 400 bp amplifiable fragments.
(XLSX)
