# Supplementary figures and images for: High-Throughput Amplicon-Based Copy Number Detection of 11 Genes in Formalin-Fixed Paraffin-Embedded Ovarian Tumour Samples by MLPA-Seq
Source: PLoS One. 2015 Nov 16;10(11):e0143006. doi: 10.1371/journal.pone.0143006 (PMC4646639; doi:10.1371/journal.pone.0143006)

Mean ratio  $\pm$  SEM

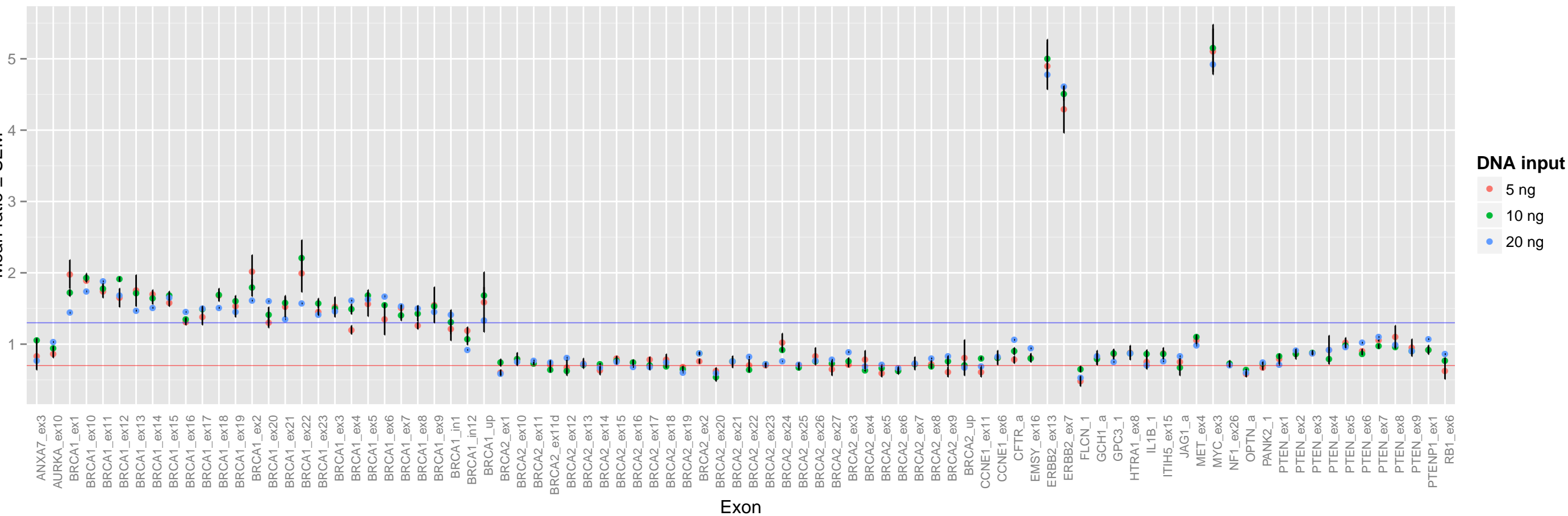

Supplement: S1 Fig — An FFPE sample with very poor quality DNA was used for the assessment, with triplicate testing for lower DNA inputs (5ng and 10 ng). (PDF) [file pone.0143006.s001.pdf]
